# Effect of curcumin on rat sperm morphology after the freeze-thawing process

**Published:** 2013

**Authors:** Ali Soleimanzadeh, Adel Saberivand

**Affiliations:** *Department of Clinical Sciences, Faculty of Veterinary Medicine, Urmia University, Urmia, Iran.*

**Keywords:** Curcumin, Rat, Sperm parameters

## Abstract

Reactive oxygen species (ROS) generation, induced by the cryopreservation process, can be responsible for mammalian sperm damage. Curcumin is known as an effective antioxidant against oxidative stress. The aim of this study was to evaluate the effects of curcumin on sperm count, motility and viability, semen total antioxidant capacity and DNA integrity of rat spermatozoa during semen freeze-thawing process. Sperm collected from 10 adult rats were divided into two groups (n=10 for each group): control and a test group supplemented with 2.5 mM curcumin. After freezing-thawing, the number of spermatozoa, motility, viability, total antioxidant capacity (TAC), and DNA integrity of the sperm were analyzed. Motility, viability and DNA integrity of sperm were significantly preserved in treatment groups compared to the control (*p* < 0.05) after freezing-thawing. Following cryopreservation, TAC was significantly preserved in thawing semen supplemented with curcumin compared to the control group (*p*<0.05). Based on our results, it is concluded that curcumin addition during freezing resulted in positive effects on sperm parameters after thawing in adult rats.

## Introduction

Semen freezing and thawing procedures produce membrane damage that reduce longevity of sperm in the female genital tract, with a concomitant decrease in fertility.^1^ The success of semen freezing relies upon an understanding of internal (inherent characteristics of spermatozoa) and external (composition of diluents, type and concentration of cryoprotective agents, rates of dilution and cooling, equilibration, and method of freezing and thawing of semen) factors and their interactions which can influence the capacity of sperm to survive during freezing and thawing.^2^

Reactive oxygen species (ROS) generation, induced by cryopreservation process, can be responsible for mammalian sperm damage.^3^^-^^5^ The ROS production has been associated with reduction of sperm motility, decreased capacity for sperm-oocyte fusion and infertility. Against ROS attack, sperm cells are well equipped with a powerful defense system of antioxidants, but an imbalance between the production of ROS and the available antioxidant-defenses results in oxidative stress.^6^ A variety of antioxidants have been tested to either scavenge ROS directly or effects of counter ROS toxicity in semen of a variety of mammalian and avian species.^7^

Antioxidants are the frontline of defense against free radicals.^8^ Curcumin (1,7-bis [4-hydroxy-3-methoxyphenyl]-1,6-heptadiene-3,5-dione) is the principal curcuminoid found in turmeric and is generally considered as its most active constituent.^9^^,^^10^ Turmeric exhibits anti-tumor, anti-infectious and anti-inflammatory activities with low toxicity.^11^^-^^13^ The most important feature of curcumin is that it has no side effects despite being a therapeutic agent with multiple beneficial functions.^14^ Curcumin is considered to be an effective antioxidant against oxidative tissue damage. The antioxidant mechanism of curcumin is due to its specific conjugated structure of two methoxylated phenols and an enol form of β-diketone. This structure is responsible for free radical trapping ability as a chain breaking antioxidant.^15^ It can significantly inhibit generation of ROS both *in vitro* and *in vivo*.^16^ Protective effects of curcumin on the testis has been reported in oxidative damage induced by cisplatin, sodium arsenite, aflatoxin, ischemia-reperfusion injury, di-n-butyl phthalate in laboratory animals.^12^^,^^17^^-^^20^

Several numbers of studies have established the effectiveness of antioxidants like curcumin against oxidative stress.^21^^-^^24^ Curcumin is reported to protect male reproductive system in chromium-induced oxidative damage.^25^ The aims of this study were to evaluate the effects of curcumin on sperm count, motility, viability, semen TAC and DNA integrity of rat spermatozoa supplemented with curcumin during semen freeze-thawing process.

## Materials and Methods

All chemicals used in the experiment were purchased from Sigma-Aldrich, Munich, Germany unless otherwise stated.

**Experimental animals. **Ten adult sexually mature male (4 months of age weighing 200-230 g) albino Wistar rats were obtained from animal house of the Faculty of Veterinary Medicine, Urmia University. They were housed in a specific pathogen-free environment under standard conditions of temperature (23 ± 1 ˚C), relative humidity (55 ± 10%), and 12/12 hr light/dark cycle, and fed with a standard pellet diet and water. All ethical themes of the studies on animals were considered carefully.

**Semen collection**. Epididymal sperms were collected by slicing the cauda region of the epididymis in 5 mL of HTF and incubated for 5 min at 37 ˚C in an atmosphere of 5% CO_2_ to allow sperm to swim out of the epididymal tubules. After collection, semen samples were divided into two groups (n=10 for each group). One control group without antioxidants and one test group supplemented with 2.5 mM curcumin^26^. All samples were packed in 0.5 mL French straws and were frozen in liquid nitrogen vapor, (4 cm above the liquid nitrogen), for 15 min and then plunged into liquid nitrogen for storage. The frozen straws were then thawed individually (37 ˚C for 20 sec) in a water bath. In frozen thawed semen samples, the sperm motility, count, live percent, DNA integrity and semen total antioxidant capacity (TAC) were evaluated using conventional methods.

**Sperm motility. **Assessment of sperm motility was done according to WHO laboratory manual protocol for the examination of human semen and sperm-cervical mucus interaction.^27^ In brief, 10 μL of the sperm suspension was placed on semen analysis chamber. A minimum of five microscopic fields were assessed to evaluate sperm motility on at least 200 sperm for each animal. 

**Sperm viability. **Eosin-nigrosin staining was used to assess sperm viability according to WHO protocol. Briefly, eosin (Merck, Darmstadt, Germany) and nigrosin (Merck, Darmstadt, Germany) was prepared in distilled water. One volume of sperm suspension was mixed with two volume of 1% eosin. After 30 sec, an equal volume of nigrosin was added to this mixture. Thin smears were then prepared and observed under a light microscope (Model CHT, Olympus optical Co. Ltd., Tokyo, Japan) at 1000× magnification. Viable sperm remained colorless while nonviable sperm was stained red. 

**Sperm morphology. **For the analysis of morphological abnormalities, sperm smears were drawn on clean and grease-free slides, and allowed to air-dry overnight. The slides were stained with 1% eosin-Y/5% nigrosin and examined at 400× for morphological abnormalities in each sample.

**Assessment of DNA integrity. **Thin smears were prepared from the sperm solution and allowed to air-dry. To test sperm DNA integrity, the smears were stained with acridine orange (AO). The AO staining was performed according to a protocol described by Tejada *et al*.^28^ In brief, the smears were fixed for 14 hr in methanol/acetic acid (3:1) at 4 ˚C and stained with AO solution (0.19% in phosphate citrate buffer, pH = 2.5) for 10 min. The slides were gently washed by distilled water for 5 min and air dried. The stained smears were then observed under fluorescence microscope (Model GS7, Nikon Co., Tokyo, Japan) at 1000× magnification. Three types of staining patterns were considered in sperm head; green spermatozoa (double-stranded DNA), yellow and red spermatozoa (single-stranded DNA). At least 100 spermatozoa per slide were counted to evaluate the percentage of double-stranded DNA in the spermatozoa. 

**Total antioxidant capacity evaluation. **The TAC content of the seminal plasma was determined by the ferric reducing antioxidant power (FRAP) assay.^29^ In brief, 300 mmol L^-1^ acetate buffer of pH 3.6, 10 mmol L^-1^ 2,4,6‐tri‐(2‐pyridyl)‐1,3,5‐triazine, 98.00% and 20 mmol L^-1^ FeCl_3_·6H_2_O were mixed together in the ratio of 10:1:1, respectively, to give the working FRAP reagent. A 50 µL aliquot of sperm samples was added to 1 mL of FRAP reagent in a semi‐micro plastic cuvette. Absorbance measurement was taken at 593 nm (A593) exactly 10 min after mixing using 50 µL of water as the reference. To standardize, 50 µL of the standard (FeSO_4_·7H_2_O, 1 mmol L^-1^) was added to 1 mL of FRAP reagent. 

**Statistical analysis. **The data analysis was performed by analysis of variance (ANOVA) and Duncan tests using SPSS (Version 17 SPSS Inc., Chicago, IL, USA). A value of *p* < 0.05 was considered as statistically significant. 

## Results

**Sperm count. **There was no significant difference between sperm number in curcumin group compared to that of the control group (Table 1).

**Sperm motility**. The sperm motility was significantly (*p *< 0.05) preserved in curcumin group compared to that of the control group (Table 1). 

**Fig. 1:**
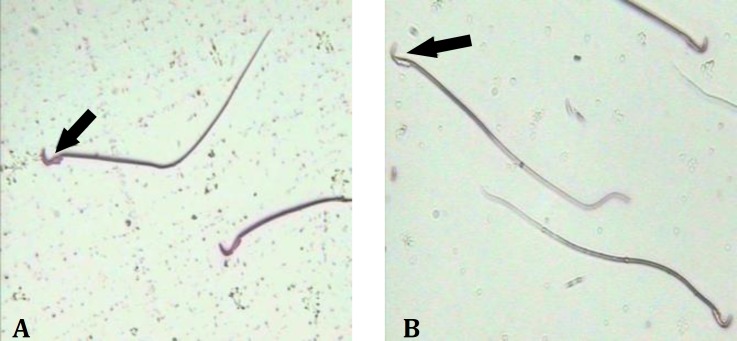
Sperm viability in curcumin group; **A)** Dead sperm (red), **B)** Viable sperm (colorless), (Black arrows); (Eosin/nigrosin, 1000×).

**Sperm viability. **The percentage of viable sperm was significantly (*p* < 0.05) preserved in curcumin group compared to that of the control group (Fig. 1 and Table 1).

**Sperm morphological anomalies. **Spermatozoa stained with eosin-nigrosin showed that addition of curcumin had no significant effect on the sperm morphology compared to that of the control group (Table 1).

**Fig. 2:**
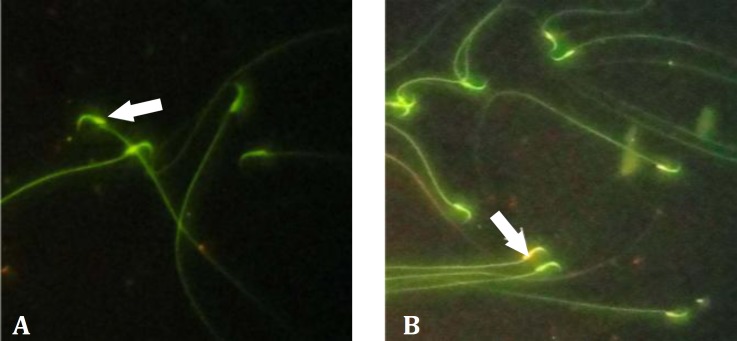
Rat spermatozoa; **A)** Normal sperm (green), **B)** Damaged DNA (yellow), (White arrows); (AO, 1000×).

**Sperm DNA integrity**. Thawed spermatozoa stained with AO showed that addition of curcumin had a significant effect on the sperm DNA integrity compared to that of the control group (Fig. 2 and Table 1). 

**Sperm Total antioxidant capacity (TAC). **The TAC of sperm was significantly (*p *< 0.05) preserved in curcumin group compared to that of the control (Table 1). 

**Table 1.** Sperm number, viability, morphological anomalies, motility, TAC and DNA integrity (AO staining) in frozen-thawed rat semen. Data are presented as mean ± SD (n = 10).

**Sperm characteristics**	**Control**	**Curcumin**
**Number (10** ^6^ **)**	20.92 ± 0.14	20.96 ± 0.24
**Viability (%)**	66.07 ± 0.51	72.96 ± 0.09 *
**Morphological anomalies (%)**	1.21 ± 0.01	1.20 ± 0.01
**Motility (%)**	67.58 ± 0.34	73.61 ± 0.44 *
**DNA integrity (%)**	80.08 ± 0.27	99.19 ± 0.19 *
**TAC ** **(μmol L** ^-1^ **)**	62.32 ± 0.17	70.16 ± 0.26 *

* indicates significantly different from controls (*p* < 0.05).

## Discussion

Cryopreservation increases the percentage of dead or membrane damaged sperm.^30^ The process of cryo-preservation is known to cause more production of reactive oxygen species, as antioxidant defenses are reduced in the process.^31^ Hence, addition of antioxidants may have beneficial effects on spermatozoa function during the cryopreservation. Curcumin has been shown to have strong antioxidant activity and reduces oxidative stress.^23^^,^^24^ The present study reports the ameliorative effects of curcumin on the motility, viability, TAC and DNA integrity of rat frozen-thawed sperm. 

The spermatozoon motility and viability generally declines following cryopreservation procedure. There is a remarkable decrease in viability and motility caused by ROS resulting in low conception rate following the use of frozen-thawed sperm for artificial insemination.^32^ Based on our results, in frozen-thawed semen, curcumin addition had a positive impact on both motility and viability. One of the possible ameliorative mechanisms of curcumin on the above mentioned parameters is to scavenge the free radicals and thereby act as good antioxidants. Another reason for enhancement of sperm motility in frozen semen observed in this study may be due to the increasing of TAC level of semen in curcumin supplemented group and the correlation between total antioxidant capacity in seminal plasma and sperm motility.^33^ These results were in agreement with the findings of earlier studies on the ameliorative effect of curcumin on the spermatozoa motility in metronidazole-treated mice.^34^

The integrity of sperm DNA is an important factor for the success of fertilization as well as normal development of the embryo, fetus and newborn.^35^ Several studies have shown the effect of cryopreservation procedure on DNA damages through induction of oxidative stress and the generation of ROS.^36^^,^^37^ In this study, a significant pre-servation of DNA integrity in post-thaw in curcumin supplemented group compared to the control, was most probably due to increase in its antioxidant capacity.

The TAC in seminal plasma is closely related to male fertility, appropriate TAC provides a favorable environment for sperm swimming. The decreased level of TAC in seminal plasma may be one of the causes of male infertility.^33^ In addition, Contri *et al*. reported a positive correlation between sperm parameters and total antioxidant capacity in seminal plasma.^38^ Semen processing and cryopreservation decrease the antioxidant defense capacity of semen. Based on our results addition of curcumin to fresh sperm of rats before freezing, significantly (*p *< 0.05) preserved the TAC level in thawing seminal plasma. 

Based on our results, it is concluded that addition of curcumin during cooling, resulted in positive effects on rat sperm motility, viability, DNA integrity and TAC level after thawing .Such data could help to develop and improve semen handling and storage techniques. Future research should address molecular mechanisms underlying the protective effects of curcumin on rat sperm. 
